# Composition of Overlapping Protein-Protein and Protein-Ligand Interfaces

**DOI:** 10.1371/journal.pone.0140965

**Published:** 2015-10-30

**Authors:** Ruzianisra Mohamed, Jennifer Degac, Volkhard Helms

**Affiliations:** 1 Center for Bioinformatics, Saarland University, Building E2 1, P.O. Box 151150, 66041, Saarbruecken, Germany; 2 Department of Pharmaceutical Life Sciences, Faculty of Pharmacy, Universiti Teknologi MARA (UiTM), 42300 Puncak Alam, Selangor, Malaysia; Universität Erlangen-Nürnberg, GERMANY

## Abstract

Protein-protein interactions (PPIs) play a major role in many biological processes and they represent an important class of targets for therapeutic intervention. However, targeting PPIs is challenging because often no convenient natural substrates are available as starting point for small-molecule design. Here, we explored the characteristics of protein interfaces in five non-redundant datasets of 174 protein-protein (PP) complexes, and 161 protein-ligand (PL) complexes from the ABC database, 436 PP complexes, and 196 PL complexes from the PIBASE database and a dataset of 89 PL complexes from the Timbal database. In all cases, the small molecule ligands must bind at the respective PP interface. We observed similar amino acid frequencies in all three datasets. Remarkably, also the characteristics of PP contacts and overlapping PL contacts are highly similar.

## Introduction

Protein-protein interactions (PPIs) play major roles in many biological processes such as bioenergetics, immune response, signal transduction, structural organization, and apoptosis [1,2]. Recently, PPIs also became a promising new target for therapeutic intervention. Unlike established pharmaceutical efforts that are directed, for example, at enzymes, G-protein coupled receptors (GPCR), or ion-channels, PPIs are challenging subjects because there are usually no convenient natural substrates that can be exploited as starting points for small-molecule design. Moreover, the lack of information about particular interface residues determining the affinities and specificities at such interfaces makes it quite hard to design compounds that are capable of interfering with PPIs. Hence, there is a strong need to characterize the properties of protein interfaces that may also bind small-molecule ligands and the underlying molecular principles of contacts they are involved in.

The Protein Data Bank (PDB) [3] is the primary resource for elucidating the diversity of atomic contacts in protein-protein (PP) and protein-ligand (PL) interactions. Many statistical analyses of molecular interactions have been done based on this resource [1, 4–6]. Furthermore, some secondary databases that are derived from the PDB have been created to assist the integrated research on PP and PL interactions. Examples for this are the Timbal database (http://mordred.bioc.cam.ac.uk/timbal) which stores data of small molecules modulating protein—protein complexes [7], the Mother of All Database (MOAD) which contains data on ligand-protein binding (http://bindingmoad.org) [8–9], the 2P2I database of structures of PP complexes with known small molecule inhibitors (http://2p2idb.cnrs-mrs.fr) [10], the Analysing Biomolecular Contacts (ABC) database (http://service.bioinformatik.uni-saarland.de/ABCSquareWeb/) [11], and the database of structurally defined protein interfaces named PIBASE (http://pibase.janelia.org/pibase2010/queries.html) [12]. One important aim in interface analysis is to identify properties which may distinguish binding residues from the rest of the protein surfaces.

Although protein-protein interfaces are rather large, planar and well packed depending [1,13], some parts of these interfaces termed overlap or bifunctional regions may bind both to small-molecule ligands and to proteins. The remaining regions of the interface which bind only to either protein or ligand are called non-overlap or monofunctional regions. Davis and Sali [14] found that bifunctional regions were enriched in tyrosine and tryptophan residues and depleted from alanine, isoleucine, leucine and valine when compared to monofunctional positions. Walter et al. [15] found for a different dataset that the overlap regions were mostly found in pockets and some of their surfaces were exposed to the solvent. Koes and Camacho [16] used Small Molecular Inhibitor Starting Points (SMISPs) from PL and PP complexes in the PDB to train statistical classifiers for predicting such SMISPs.

In this study, we analyzed the residue-residue and atomic contact frequencies and propensities of five non-redundant datasets i) 174 protein-protein and ii) 161 protein-ligand complexes from Walter [15], iii) 436 protein-protein and iv) 196 protein-ligand complexes from the PIBASE database [12], and v) a dataset of 89 protein-ligand complexes from the Timbal database [7]. Our main research question was to find out whether small molecule ligands have similar physio-chemical features as protein binding interfaces when they bind at overlapping PP/PL binding interfaces and this was indeed found to be the case.

## Material and Methods

### Datasets

Non-redundant datasets from three different databases were used to investigate the composition of protein interfaces. The first pair of datasets consists of 174 PP complexes and 161 PL complexes compiled by Walter et al. [15] from the ABC database [11] (see Tables A and B in S1 File). 25 entries of this PL dataset had been updated in the PDB in the meantime. We changed 22 previous ligand names to the current ligand names in the PDB files and removed 14 PDB files because they contain modified residues that were wrongly recognized as ligands before [15]. As described by Walter et al. [15], these complementary PP and PL datasets fulfill the following criteria: (i) PP: PL pairs represent pairs of complexes, where one protein may bind either a second protein or a small molecule ligand at the same interface, (ii) every pair of the dataset is represented as (Pi1, Pi2): (Pi3, Lj), where Pi1, Pi2 and Pi3 are three proteins and Lj is a small molecule ligand, (iii) Pi1 and Pi3 share at least 40% sequence identity, and (iv) the aligned positions in the binding interfaces of Pi1–Pi2 and Pi3 –Lj have at least two residues in common.

The same criteria of (Pi1, Pi2): (Pi3, Lj) pairs of PP and PL complexes from Walter et al., were then applied to the datasets of PP and PL complexes from the PIBASE database [12]. To avoid redundancy among these complexes, we clustered the PL complexes using the CD-Hit program [17,18] with the same sequence identity cut-off of 40%. Within a cluster, we selected the representative PP:PL pair with the highest identity score of the interface residues. Additionally, we discarded clusters which contained only sequences with fewer than 40 amino acids. The final pair of datasets comprises 436 PP complexes (Table C in S1 File) and 196 PL complexes (Table D in S1 File).

Interactions where both interacting chains have > 90% sequence identity are defined as homodimer complexes and the remainder as heterodimer complexes. As a result, the PP complexes from the ABC dataset comprised 94 homodimer complexes and 80 heterodimer complexes (see Tables A and B in S2 File). The PP complexes from the PIBASE dataset were grouped into 335 homodimer complexes and 101 heterodimer complexes (see Tables C and D in S2 File).

The fifth dataset was extracted from the table of PDB entries in the Timbal database (see Table E in S1 File). First, the 1695 entries in the current version of the Timbal database were filtered by removing complexes containing ligands that are annotated to act as stabilizers. Then, the CD-Hit program was applied to remove redundancy among the protein chains of the complexes with the sequence identity cut-off of 40%. We also eliminated clusters of proteins with fewer than 40 amino acids. This gave a final dataset of 89 protein-small molecule complexes.

Data from the ABC, PIBASE, and Timbal databases was retrieved by using MySQL queries, Java, Biojava [19] and analyzed with the R software (http://www.R-project.org).

### Surface and Interface Residues

**Fig 1:**
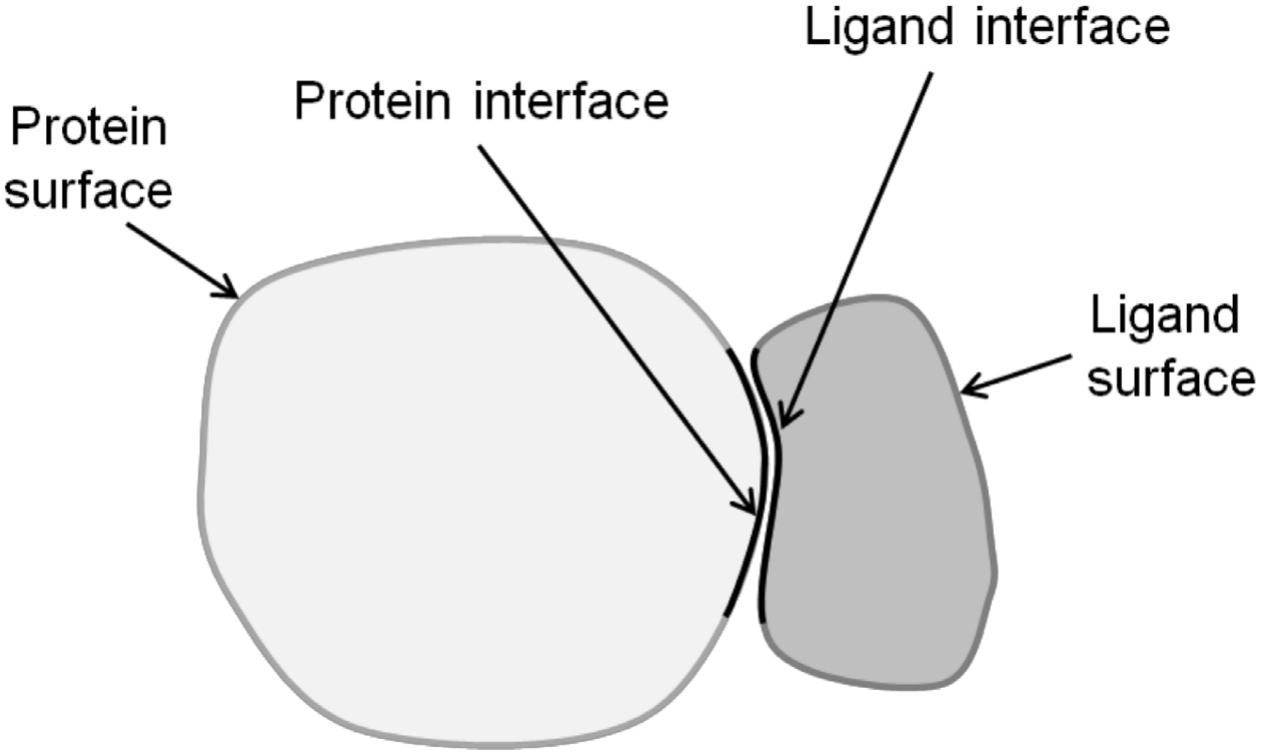
Schematic illustration of a PL complex illustrating the interface (black border) and the remaining surface regions. PL, protein-ligand.

The solvent accessible surface area (SASA) was calculated using the NACCESS program [20]. As surface residues we considered those residues with a SASA value larger than zero. Labeled as interface residues were those residues that are within a radius of either 3 Å, 4 Å or 5 Å of any residue of the binding partner. Fig 1 shows a schematic diagram how we determined the interface and the remaining surface of PL complexes.

### Classification of the amino acids

The standard classification according to the Eisenberg hydrophobicity scale [21] was used to classify amino acids into four categories: hydrophobic (Ala, Ile, Leu, Met, Phe, Pro, Val), charged (Arg, Asp, Glu, Lys), polar (Cys, Asn, Gln, His, Ser, Thr, Trp, Tyr), and Gly.

### Interface Residue Propensities

Residue interface propensities were calculated for the homodimeric and heterodimeric protein-protein complexes of the ABC and PIBASE datasets and for the protein-ligand complexes of the ABC, PIBASE and Timbal datasets. These propensities give a measure of the relative importance of different amino acid residues in the interface, compared with the surface as a whole. The propensities were calculated with the following formula:

$$Interface\;residue\;propensity\;AAj=\left(\frac{\sum interface\;residues\;of\;type\;j}{\sum all\;interface\;residues}\right)/\left(\frac{\sum surface\;residues\;of\;type\;j}{\sum all\;surface\;residues}\right)$$

An interface residue propensity of >1.0 indicates that a residue type occurs more frequently in interfaces than on the protein surface in general.

### Contacts between amino acids of the two proteins

For every PP complex, we counted the observed number of contacts between amino acids of the first protein and amino acids of the second protein. A contact exists between two residues of these proteins if any residue of the first protein is within a distance threshold of 5.0 Å from the other protein. This was represented in a 20 x 20 table. From the 400 observed counts of amino acid pairs in the two datasets of protein-protein complexes, we derived normalized pair frequencies with the following formula:

$$Normalization=\frac{\left(\frac{\sum contacts\;of\;residue\;pair\;XY}{\sum all\;residue\;contacts}\right)}{\left(\frac{\sum observed\;X\;on\;surface}{\sum all\;surface\;residues\;of\;first\;protein}\right)\left(\frac{\sum observed\;Y\;on\;surface}{\sum all\;surface\;residues\;of\;second\;protein}\right)}$$

Here, XY is the number of observed contact pairs between residues X and Y across the interface, X is the count of amino acid X in the first protein and Y is the count of amino acid Y in the second protein.

### Atom contacts in protein-protein and protein-ligand complexes

In protein-protein and protein-ligand complexes, we considered two surface atoms belonging to separate molecules to be in contact and labeled them as interface atoms if the distance between them is less than 5.0 Å. We counted contacts between all pairs of carbon (C), fluorine (F), nitrogen (N), oxygen (O), phosphorus (P), and sulfur (S) atoms resulting in 36 contact pairs. Then, the absolute counts were normalized as follows:

$$Normalization=\frac{\left(\frac{\sum contacts\;of\;atom\;pair\;AB}{\sum all\;atom\;contacts}\right)}{\left(\frac{\sum observed\;A\;on\;surface}{\sum all\;surface\;atom\;of\;ligand\;or\;protein}\right)\left(\frac{\sum observed\;B\;on\;surface}{\sum all\;surface\;atom\;of\;protein}\right)}$$

where A is the count of atom type A in the first protein (PP complexes) or protein (PL complexes), B is the count of atom type B in the second protein (PP complexes) or ligand (PL complexes) and, AB is the number of observed contact pairs between atom types A and B across the interface.

According to Higueruelo et al. [22], atom type contacts were grouped into polar and apolar contacts as follows: For protein-protein complexes, apolar contacts exist between C…C, C…S and S…S (not in Cys-Cys bridges). Polar contacts involve the pairs N…O, O…O, N…N, O…S and N…S (from Cys). For protein-ligand complexes, apolar contacts are C…C, and C…S pairs whereas polar contacts are formed by the pairs N…O, O…O, N…N, O…S, N…S, N…F, O…F, and S…F (from Cys).

### Calculation of polarity ratio and interface atom ratio

The polarity ratio (PR) is a simple measure of the polarity of the interface [23]. It was defined as the ratio of the number of polar atoms N, O, S at the interface to the sum of all C, N, O, S at the interface.

The interface atom ratio (IR) is a measure for the fraction of surface atoms that are located at the interface. It was calculated for the interfaces of protein-protein and protein-ligand complexes. Only the six heavy atom types C, N, O, S, P and F were considered in the calculation. IR is the ratio of the sum of all atoms at the interface to the sum of all atoms at the surface.

## Results and Discussion

**Fig 2:**
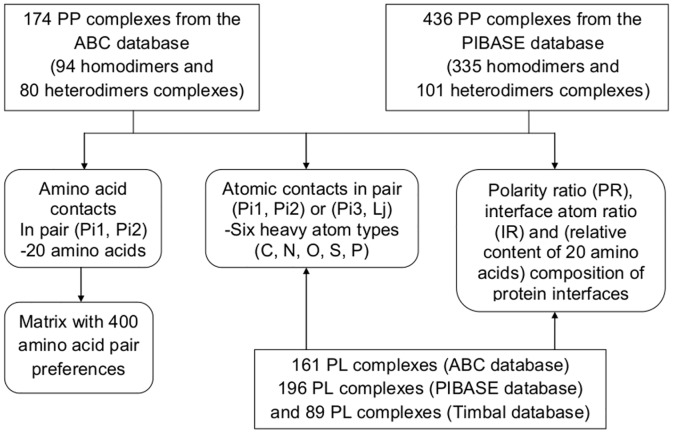
Flow chart summarizing the compilation of contacts between amino acids of the first protein (Pi1) and amino acids of the second protein (Pi2), atom contacts in PP and PL complexes, and the calculation of PR and IR. PP, protein-protein; PL, protein-ligand; PR, polarity ratio; IR, interface atom ratio.

PPI interfaces are known to possess particular geometric and physicochemical characteristics, see e.g. [1,24–26]. Comparing these features of protein interfaces to those of overlapping protein-ligand interfaces should aid in targeting protein-protein interaction sites. Here, we used the ABC, PIBASE and Timbal databases as data sources for protein interfaces and surfaces. All three databases are secondary database that are derived from the PDB. However, due to the different way of identifying overlapping PP/PL pairs, the direct overlap between the three non-redundant datasets derived from them is fairly small. We believe that this may have resulted from the clustering with the CD-Hit program that selected different cluster representatives in each case. We found only the following redundant PP complexes 1AB8 (B-A), 1AZZ (A-C), 1BMF (F-B), 1EYS (H-M), 1RQ8 (A-E), 1SGF (G-B) from the ABC dataset and 1AB8 (A-B), 1AZZ (C-A), 1BMF (C-D), 1EYS (M-C), 1RQ8 (E-A), 1SGF (G-Z) from the PIBASE dataset. Furthermore, both datasets share the following lists of PDB IDs with same chain interactions 1DPJ (A-B), 1P0S (H-E), and 2G2U (A-B). Similarly, there are few redundancies between datasets of PL complexes from ABC and PIBASE, namely 1C50 (A-CHI), 1KYN (A-KTP), 1LBC (A-CYZ) and 1M2Z (A-BOG), respectively. There is also one overlapping member between the datasets of PL complexes from PIBASE and Timbal, namely the PDB ID 1AB8 (A-FOK). Fig 2 summarizes the workflow of the analysis of the five datasets. The fraction of homodimers and heterodimers in the datasets derived from ABC and PIBASE are 54%: 46% and 77%: 23%, respectively.

### Amino acid composition and protein interfaces propensity

**Fig 3:**
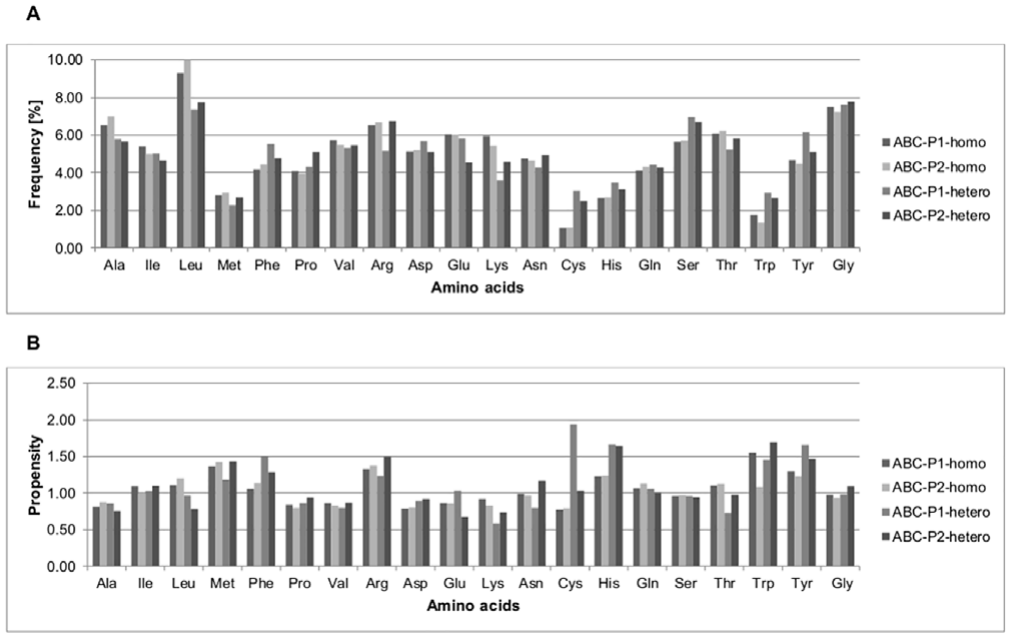
Percentage frequencies and propensities of amino acid residues at protein interfaces of PP complexes from the ABC dataset. (A) Percentage frequencies of amino acid residues at protein interfaces. (B) Propensities of amino acid residues at protein interfaces. PP, protein-protein; ABC, ABC dataset; homo, homodimeric PP interface; hetero, heterodimeric PP interfaces; P1, protein interface of the first protein (Pi1); P2, protein interface of the second protein (Pi2).

**Fig 4:**
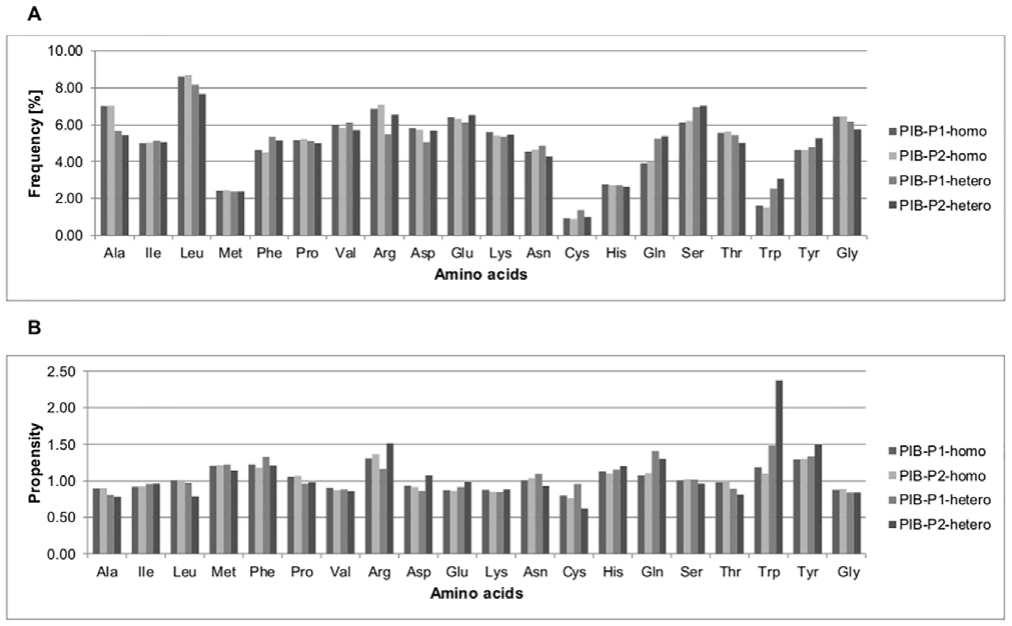
Percentage frequencies and propensities of amino acid residues at protein interfaces of PP complexes from the PIBASE dataset. (A) Percentage frequencies of amino acid residues at protein interfaces. (B) Propensities of amino acid residues at protein interfaces. PP, protein-protein; PIB, PIBASE dataset; homo, homodimeric PP interface; hetero, heterodimeric PP interfaces; P1, protein interface of the first protein (Pi1); P2, protein interface of the second protein (Pi2).

**Fig 5:**
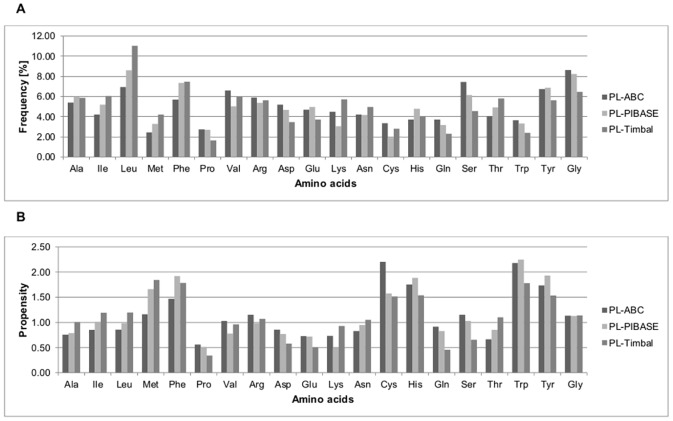
Percentage frequencies and propensities of amino acids residues at protein interfaces of PL complexes from the ABC, PIBASE and Timbal datasets. (A) Percentage frequencies of amino acids residues at protein interfaces. (B) Propensities of amino acids residues at protein interfaces. PL, protein-ligand; PL-ABC, PL complexes from the ABC dataset; PL-PIBASE, PL complexes from the PIBASE dataset; PL-Timbal, PL complexes from the Timbal dataset.

Figs 3 and 4 show the percentage frequencies and propensities of amino acids at the interfaces of homodimeric and heterodimeric PP complexes from the ABC and PIBASE datasets, respectively. Fig 5 shows the percentage frequencies and propensities of amino acids at the protein interfaces of the PL complexes from the ABC, PIBASE and Timbal datasets, respectively. Previous studies showed that protein-protein interfaces have unique characteristics that distinguish them from non-interface portions of protein surfaces [24,27,28]. By grouping the amino acids according to the Eisenberg hydrophobicity scale (see methods) we found that, hydrophobic amino acids account for 38.06% (ABC-P1-homo), 38.87% (ABC-P2-homo), 38.81% (PIB-P1-homo) and 38.75% (PIB-P2-homo) at interfaces of homodimeric PP complexes compared to 35.60% (ABC-P1-hetero), 36.11% (ABC-P2-hetero), 37.94% (PIB-P1-hetero) and 36.38% (PIB-P2-hetero) at interfaces of heterodimeric PP complexes (Figs 3A and 4A). This matches the general finding e.g. of Jones and Thornton who stated that homodimer complexes are more hydrophobic [1]. At interfaces of both homodimeric and heterodimeric PP complexes from the ABC and PIBASE datasets, alanine, valine, and lysine residues are underrepresented with propensities lower than 1.0 (Figs 3B and 4B). One hydrophobic amino acid (leucine), one charged amino acid (lysine) and two polar amino acids (glutamine and threonine) have higher propensities at interfaces of homodimer complexes than at interfaces of heterodimer complexes of the ABC dataset. In the PIBASE dataset, four hydrophobic amino acids (alanine, leucine, proline and valine), one polar amino acid (threonine) and glycine have higher propensities in homodimer complexes than in heterodimer complexes.

As expected, hydrophobic and polar residues make up the largest portion of protein interfaces. In fact, this is one of the challenges for targeting PPIs with small molecules as the contact surfaces between proteins typically involve many hydrophobic and polar interactions distributed over a large interface with buried area of ~1500–3000 Å^2^ [29]. According to the classification by Eisenberg, the fractions of hydrophobic, polar, charged and glycine residues are 36.95%, 33.38%, 22.11%, 7.56% for the first protein (Pi1), 37.70%, 32.48%, 22.35%, 7.46% for the second protein (Pi2) of the PP complexes from the ABC dataset, 38.60%, 30.93%, 24.09%, 6.38% for the first protein (Pi1), and 38.20%, 31.35%, 24.22%, 6.23% for the second protein (Pi2) of the PP complexes from the PIBASE dataset. Although there are minor differences between the two datasets (slightly more charged and fewer glycine residues in the PIBASE dataset), we found the composition to be overall remarkably similar.

At the interfaces of both homodimeric and heterodimeric PP complexes from the ABC and PIBASE datasets, the frequencies of methionine and tryptophan at protein interfaces are at most 3.07%. However, both amino acids have normalized interface propensities clearly larger than one, suggesting that these residues play important roles and thus occur more frequently at protein interfaces rather than elsewhere on the protein surface. Overall, tryptophan, tyrosine and arginine each have propensities above 1.0 at both protein interfaces of homodimeric and heterodimeric PP complexes from the ABC and PIBASE datasets. This reflects that aromatic amino acids and arginine play important roles in protein interfaces, which is a well-known fact. For example, Bogan and Thorn [30] reported that hotspot regions at protein interfaces are enriched in tryptophan, tyrosine and arginine. Also, Jones, Marin and Thornton [31] found that hydrophobic residues including tryptophan and tyrosine as well as arginine are moderately enriched at protein interfaces compared to the whole surface. Jones and Thornton [1] reported that with the exception of methionine, all hydrophobic residues show a greater preference for the interfaces of homodimers than for those of heterocomplexes. Based on our analysis, only leucine is clearly enriched at homodimer interfaces. Janin, Bahadur and Chakrabarti [26] wrote that relative to the accessible protein surface, the interfaces are depleted in glutamic acid, aspartic acid, and lysine, and enriched in methionine, tyrosine and tryptophan. Our findings are in good agreement with this. In our case, the enriched category also includes phenylalanine, histidine and arginine. The underrepresented category also includes alanine, proline and valine. Talavera et al. [32] provided a rather recent compilation of amino acid frequencies and propensities, separately for homomeric and heterodimeric PP complexes. A possible concern about their work is that they applied a rather generous homology threshold of 80% identity. They found tyrosine, tryptophan, methionine, cysteine, phenylalanine, leucine, valine and isoleucine to be enriched at the interfaces of homo-complexes. In the case of hetero-complexes, cysteine fell out from this list. On the other hand, lysine, asparagine, aspartic acid and glutamic acid were underrepresented in homo-complexes. The same ones plus serine and glycine were found for hetero-complexes.

The distributions of the percentage frequencies and propensities of amino acids at the protein interfaces of the PP datasets derived from ABC and PIBASE were compared with the non-parametrical Friedman test as the datasets do not have a normal distribution. As suggested by the graphical representation of Figs 3 and 4, the ABC and PIBASE datasets do not differ significantly (percentage frequencies, *P-value* = 0.99 and propensities, *P-value* = 0.97).

The fractions of hydrophobic, polar, charged and glycine residues at protein binding interfaces of PL complexes are 34.08%, 36.97%, 20.31%, 8.64% (ABC dataset), 38.25%, 35.39%, 18.12%, 8.24% (PIBASE dataset) and 42.32%, 32.61%, 18.60%, 6.47% (Timbal dataset), see Fig 5A. Compared to PP interfaces, the ligand-contacting protein interfaces of the Timbal dataset contain about 5% more hydrophobic residues, and about 5% fewer charged residues. In contrast, the ligand-contacting protein interfaces from the ABC and PIBASE datasets contain 3–4% more polar residues than PP interfaces and 3–4% less charged residues.

In the PL complexes of the ABC dataset, the five amino acids with the highest propensities found at protein interfaces are cysteine (2.20), tryptophan (2.18), histidine (1.75), tyrosine (1.74), and phenylalanine (1.47). In the PL complexes of the PIBASE dataset, the most enriched ones are tryptophan (2.25), tyrosine (1.93), phenylalanine (1.92), histidine (1.89), and methionine (1.66). In the PL complexes of the Timbal dataset, methione has the highest propensity of 1.85, followed by phenylalanine (1.78), tryptophan (1.78), histidine (1.54) and tyrosine (1.53), respectively. In all datasets of PL complexes, tryptophan, phenylalanine, histidine, and tyrosine are found most often at the protein interfaces (Fig 5B) complemented by either cysteine (ABC) or methionine (PIBASE, Timbal). S3 and S4 Files list the frequencies and propensities of amino acids present at the protein interfaces, together with the sum, mean, standard deviation and standard errors for each complex in the PP and PL datasets. The distributions of percentage frequencies and propensities of amino acids acids at the protein interfaces in the datasets derived from ABC, PIBASE and Timbal did not differ significantly (percentage frequencies, *P-value* = 0.86 and propensities, *P-value* = 0.96, Friedman rank sum test).

### Amino acid contacts

**Fig 6:**
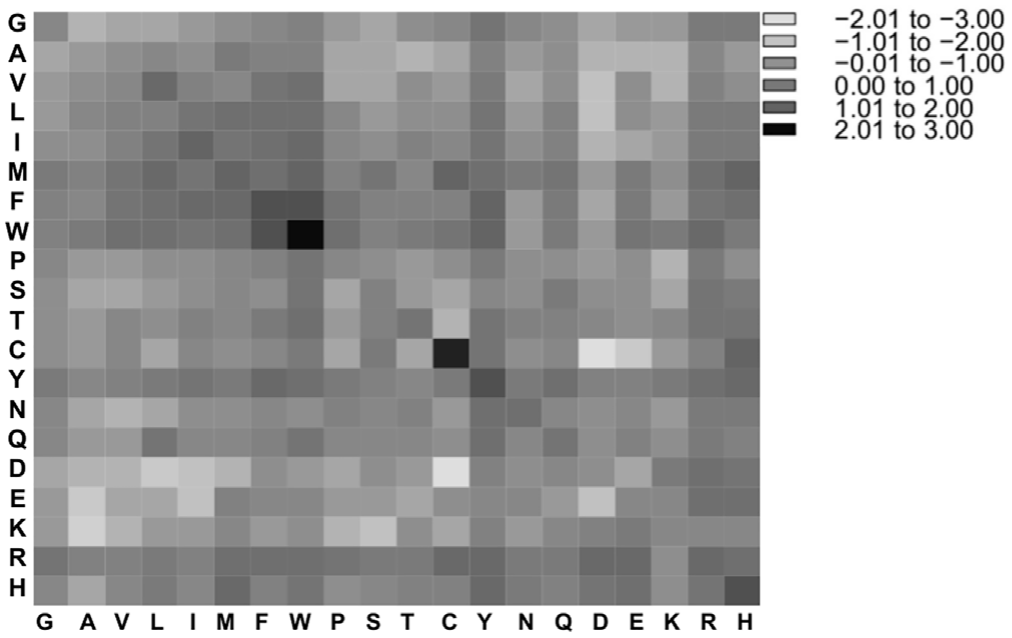
Amino acid pairing propensities (in log_2_-format) for interfaces of PP complexes from the ABC dataset. PP, protein-protein.

**Fig 7:**
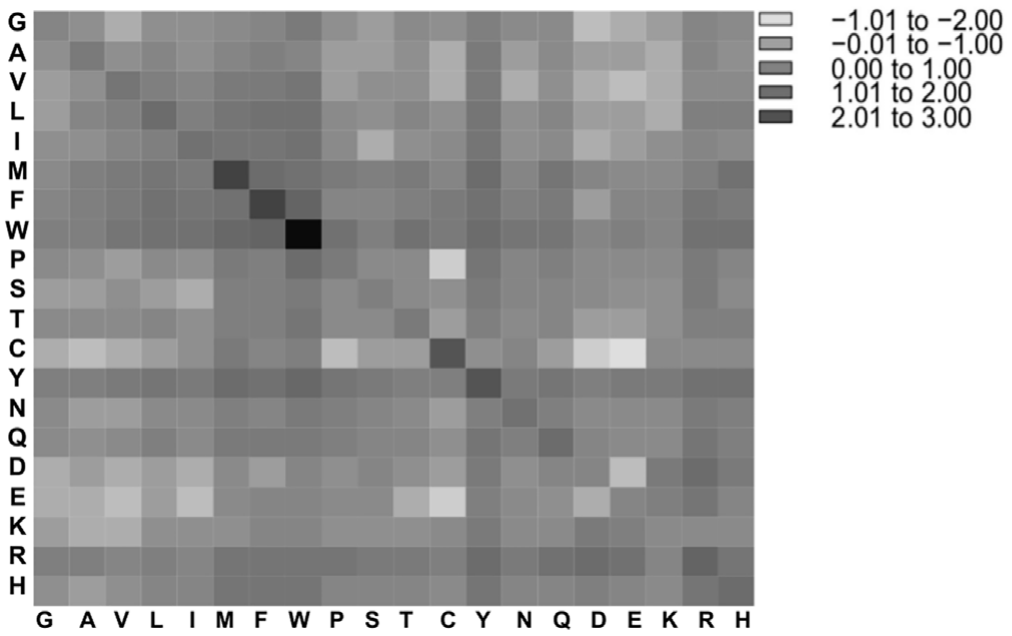
Amino acid pairing propensities (in log_2_-format) for interfaces of PP complexes from the PIBASE dataset. PP, protein-protein.

The propensities of amino acid contacts in PP complexes between amino acids of the first protein (Pi1) and amino acids of the second protein (Pi2) were obtained by counting the absolute number of contacts and normalizing this number against the appearance probability of the two involved residues at the surface. In Figs 6 and 7, the propensity values were log2 transformed to ensure a balanced view of over- and under-representation. Contacts with high propensities were observed among residues pairs of different polarity types. In PP complexes from the ABC dataset, the five most over-represented interactions were found between the pairs of tryptophan (6.32), cysteine (4.66), phenylalanine (3.61) and histidine (3.50) as well as between tryptophan and phenylalanine (3.36), see Fig 6. In PP complexes from the PIBASE dataset, the five most over-represented interactions were pairs of tryptophan (7.50), methionine (4.34), phenylalanine (3.96), tyrosine (3.57), and cysteine (3.43), see Fig 7. These results are consistent with previous studies of protein-protein interfaces that reported an enrichment of contacts between cysteine, hydrophobic contacts and aromatic contacts [24,27,33–35]. Further studies noticed that besides disulfide bonds and hydrophobic interactions, also salt-bridges contribute to stabilizing protein-protein interactions [27,33–35]. In our analysis, contacts between lysine and negatively charged amino acids (Asp, Glu) are only mildly enriched (propensity 1.23 on average), whereas those between arginine and either Asp or Glu are about two-fold enriched (2.06), see Tables A and B in S5 File, what reflects the enriched of arginine at protein interfaces.

Tables A and B in S5 File list the frequencies and propensities of amino acids contacts in PP complexes from the ABC and PIBASE datasets. The propensities of amino acid contacts between amino acids of the first protein (Pi1) and amino acids of the second protein (Pi2) in PP complexes between datasets from the ABC and the PIBASE did not differ statistically significantly (*P-value* = 0.76, Wilcoxon signed rank test).

Based on the counts of amino acids, we computed the average number of amino acid residues at the interfaces of the two proteins Pi1 and Pi2 of PP complexes and the Pi3 protein of PL complexes using three different atom distances (3 Å, 4 Å and 5 Å). At the distance threshold of 3 Å, both interfaces at Pi1 and Pi2 contain less than 10 amino acids on average. For thresholds of 4 Å and 5 Å, the average size of the protein interfaces is 26.22 (ABC dataset) and 38.69 amino acids (PIBASE dataset) (Table 1). Table 2 shows the average number of residues at the interfaces of Pi3 in PL complexes from the ABC, PIBASE and Timbal datasets. At the distance threshold of 3 Å, the average size of the interfaces is less than 3 amino acids for all datasets. At 4 Å and 5 Å atom distances, the average sizes of the interfaces are between 6.31 amino acids (ABC dataset) and 13.54 amino acids (Timbal dataset). Although the PL interfaces from the ABC dataset are clearly smaller than those from the PIBASE and Timbal datasets, the average ligand size in the ABC dataset (20.48 atoms without hydrogen atoms) is only slightly smaller than the average ligand size in the Timbal dataset (21.53 atoms) and in the PIBASE dataset (21.42 atoms), respectively.

**Table 1:** The average number with standard deviation of amino acid residues at the interfaces of PP complexes in the ABC and PIBASE datasets.

	PP complexes			
	ABC dataset		PIBASE dataset	
Atom distance	Pi1	Pi2	Pi1	Pi2
3Å	7.67 ± 6.68	7.49 ± 6.85	9.61 ± 15.62	9.53 ± 15.52
4Å	27.17 ± 19.18	26.22 ±19.7	31.13 ± 24.53	30.76 ± 24.61
5Å	34.52 ± 23.31	32.8 ± 24.11	38.69 ± 27.97	38.09 ± 27.94

PP, protein-protein; Pi1, first protein; Pi2, second protein.

**Table 2:** The average number with standard deviation of amino acid residues at the interfaces of PL complexes in the ABC, PIBASE and Timbal datasets.

	PL complexes		
Atom distance	ABC dataset	PIBASE dataset	Timbal dataset
3Å	1.64 ± 1.93	2.58 ± 2.08	2.54 ± 2.52
4Å	6.31 ± 4.66	10.04 ± 4.39	9.99 ± 6.32
5Å	8.84 ± 5.79	13.43 ± 5.63	13.54 ± 8.06

PL, protein-ligand.

### Atomic contacts in protein-protein and protein-ligand complexes

In this section, we analyzed the atomic contacts in the datasets of PP and PL complexes. For atom pairs between the first and second proteins (Pi1–Pi2) in PP complexes and between protein and ligand (Pi3 –Lj) in PL complexes, we counted contacts of less or equal to 5 Å between six types of heavy atoms, namely carbon (C), flourine (F), nitrogen (N), oxygen (O), phosphorus (P) and sulfur (S). This resulted in 36 atomic pair contacts. Table 3 lists the appearance frequency of these 36 atomic contact types in PP and PL complexes from the ABC, PIBASE and Timbal datasets. In all datasets, the most frequent contacts are C…C (> 41%), O…C (> 10%), C…O (> 8%), and C…N (> 7%).

**Table 3:** The percentage frequencies of the 36 atomic contact types in PP and PL complexes.

		PP complexes		PL complexes		
		ABC dataset	PIBASE dataset	ABC dataset	PIBASE dataset	Timbal dataset
Atom1^a^	Atom2^b^	%	%	%	%	%
C	C	44.08	44.20	41.24	44.57	43.49
C	N	10.82	10.95	8.50	9.03	7.05
C	O	10.42	10.68	12.63	11.85	8.88
C	S	0.85	0.57	0.90	0.88	1.00
C	P	0.00	0.00	0.00	0.00	0.00
C	F	0.00	0.00	0.00	0.00	0.00
N	C	8.29	8.12	5.81	5.16	5.51
N	N	2.76	2.70	1.58	1.25	1.30
N	O	2.85	2.83	2.18	1.77	1.57
N	S	0.14	0.08	0.17	0.09	0.13
N	P	0.00	0.00	0.00	0.00	0.00
N	F	0.00	0.00	0.00	0.00	0.00
O	C	10.93	11.17	13.87	12.78	15.03
O	N	4.48	4.40	4.89	4.38	6.58
O	O	3.37	3.45	4.79	4.43	4.60
O	S	0.17	0.14	0.18	0.25	0.22
O	P	0.00	0.00	0.00	0.00	0.00
O	F	0.00	0.00	0.00	0.00	0.00
S	C	0.56	0.47	0.90	1.00	0.35
S	N	0.14	0.10	0.26	0.26	0.08
S	O	0.11	0.12	0.31	0.23	0.04
S	S	0.03	0.02	0.04	0.04	0.01
S	P	0.00	0.00	0.00	0.00	0.00
S	F	0.00	0.00	0.00	0.00	0.00
P	C	0.00	0.00	0.59	0.45	1.64
P	N	0.00	0.00	0.50	0.28	1.18
P	O	0.00	0.00	0.19	0.22	0.40
P	S	0.00	0.00	0.02	0.01	0.00
P	P	0.00	0.00	0.00	0.00	0.00
P	F	0.00	0.00	0.00	0.00	0.00
F	C	0.00	0.00	0.32	0.77	0.75
F	N	0.00	0.00	0.04	0.11	0.09
F	O	0.00	0.00	0.08	0.15	0.10
F	S	0.00	0.00	0.01	0.04	0.00
F	P	0.00	0.00	0.00	0.00	0.00
F	F	0.00	0.00	0.00	0.00	0.00
**Total**		**100.00**	**100.00**	**100.00**	**100.00**	**100.00**

PP, protein-protein; PL, protein-ligand.

^a^For PP complexes, atom1 belongs to the first protein and for PL complexes, atom1 belongs to the protein.

^b^For PP complexes, atom2 belongs to the second protein and for PL complexes, atom2 belongs to the ligand.

Chen and Kurgan [36] previously characterized the binding interfaces of proteins with small molecules, irrespective of whether they also bind to other proteins. As expected, interactions with organic molecules are dominated by van der Waals contacts, hydrogen bonds, and covalent contacts, whereas those with charged species also involve electrostatic interactions. Hakulinen et al. [37] argued that small molecules frequently contact phenylalinine, histidine, tyrosine and tryptophan residues of proteins because their aromatic ring carbons prefer other aromatic carbons. Both findings match well with the results of this analysis. The atomic contacts in PP complexes of the ABC and PIBASE datasets did not differ significantly (*P-value* = 0.76, Wilcoxon signed rank test). Also the frequencies of the atomic contacts between the PL complexes of the ABC, PIBASE and Timbal datasets did not differ significantly (*P-value* = 0.11, Friedman rank sum test).

Tables 4 and 5 list the percentage frequencies and normalized propensities of apolar, polar and other atomic contacts in PP complexes and PL complexes, respectively. The content of apolar contacts (45.52% for the PP complexes in the ABC dataset and 45.25% for the PIBASE dataset) and of polar contacts (13.85% vs 13.70%) is highly similar between the two PP datasets. In contrast, the PL complexes of the PIBASE dataset (46.45%) contained more apolar contacts than the Timbal dataset (44.84%) and the ABC dataset (43.04%). Concerning polar contacts in PL complexes, the Timbal dataset (14.71%) and the ABC dataset (14.48%) contain more such contacts than the PIBASE dataset (12.95%). Overall, the differences of the normalized propensities seem minor, among the PP and PL datasets, as well as between PP and PL datasets, which agrees with the findings of [36]. In all datasets, C-C contacts are slightly overrepresented (1.04 to 1.11 times the randomly expected number of contacts). N-N contacts are always more frequent (1.07 to 1.32) than O-O contacts (0.70 to 0.95).

**Table 4:** Percentage frequencies (with normalized propensity values in parentheses) of apolar, polar and other atomic contacts of PP complexes from the ABC and PIBASE datasets.

		PP complexes	
		ABC dataset	PIBASE dataset
**Apolar contacts**:	C…C	44.08 (1.10)	44.20 (1.10)
	C…S	0.85 (2.53)	0.57 (1.91)
	S…C	0.56 (1.82)	0.47 (1.63)
	S…S (not in Cys-Cys bridge)	0.03 (10.94)	0.01 (6.60)
	**Total**	**45.52**	**45.25**
**Polar contacts**:	N…O	2.85 (0.91)	2.83 (0.90)
	O…N	4.48 (1.40)	4.40 (1.40)
	O…O	3.37 (0.70)	3.45 (0.73)
	N…N	2.76 (1.31)	2.70 (1.29)
	O…S	0.16 (1.40)	0.14 (1.38)
	S…O	0.11 (1.05)	0.12 (1.24)
	N…S (from Cys)	0.05 (0.68)	0.02 (0.35)
	S…N (from Cys)	0.07 (0.93)	0.03 (0.50)
	**Total**	**13.85**	**13.70**
**Other contacts**:	C…N	10.83 (1.19)	10.95 (1.20)
	N…C	8.28 (0.91)	8.12 (0.88)
	C…O	10.42 (0.76)	10.68 (0.78)
	O…C	10.93 (0.78)	11.17 (0.81)
	N…S (S not from Cys)	0.09 (1.14)	0.06 (0.86)
	S…N (S not from Cys)	0.08 (1.08)	0.07 (1.05)
	S…S (in Cys-Cys bridge)	0.002 (0.84)	0.003 (1.39)
	C…P/P…C	0.00 (0.00)	0.00 (0.00)
	C…F/F…C	0.00 (0.00)	0.00 (0.00)
	N…P/P…N	0.00 (0.00)	0.00 (0.00)
	N…F/F…N	0.00 (0.00)	0.00 (0.00)
	O…P/P…O	0.00 (0.00)	0.00 (0.00)
	O…F/F…O	0.00 (0.00)	0.00 (0.00)
	S…P/P…S	0.00 (0.00)	0.00 (0.00)
	S…F/F…S	0.00 (0.00)	0.00 (0.00)
	P…P	0.00 (0.00)	0.00 (0.00)
	P…F/F…P	0.00 (0.00)	0.00 (0.00)
	F…F	0.00 (0.00)	0.00 (0.00)
	**Total**	**40.63**	**41.05**
	**Grand Total**	**100**	**100**

PP, protein-protein.

**Table 5:** Percentage frequencies (with normalized propensity values in parentheses) of apolar, polar and other atomic contacts of PL complexes from the ABC, PIBASE and Timbal datasets.

		PL complexes		
		ABC dataset	PIBASE dataset	Timbal dataset
**Apolar contacts**:	C…C	41.24 (1.04)	44.57 (1.04)	43.49 (1.11)
	C…S	0.90 (2.86)	0.88 (2.82)	1.00 (2.60)
	S…C	0.90 (1.26)	1.00 (1.14)	0.35 (0.99)
	**Total**	**43.04**	**46.45**	**44.84**
**Polar contacts**:	N…O	2.18 (1.20)	1.77 (1.02)	1.57 (0.94)
	O…N	4.89 (1.31)	4.38 (1.39)	6.58 (1.68)
	O…O	4.79 (0.85)	4.43 (0.95)	4.6 (0.80)
	N…N	1.58 (1.32)	1.25 (1.07)	1.30 (1.14)
	O…S	0.18 (1.45)	0.25 (2.50)	0.22 (1.33)
	S…O	0.31 (1.22)	0.23 (0.77)	0.04 (0.36)
	N…S	0.17 (4.07)	0.09 (1.24)	0.13 (2.75)
	S…N	0.26 (1.59)	0.26 (1.30)	0.08 (0.97)
	N…F	0.00 (0.00)	0.00 (0.00)	0.00 (0.00)
	F…N	0.04 (0.56)	0.11 (0.46)	0.09 (0.88)
	O…F	0.00 (0.00)	0.00 (0.00)	0.00 (0.00)
	F…O	0.08 (0.80)	0.15 (0.43)	0.10 (0.70)
	S…F (S from Cys)	0.00 (0.00)	0.00 (0.00)	0.00 (0.00)
	F…S (S from Cys)	0.00 (0.00)	0.02 (3.21)	0.00 (0.00)
	**Total**	**14.48**	**12.95**	**14.71**
**Other contacts**:	C…N	8.50 (0.93)	9.03 (0.91)	7.05 (0.77)
	N…C	5.81 (1.12)	5.16 (1.03)	5.51 (1.13)
	C…O	12.63 (0.91)	11.85 (0.81)	8.88 (0.66)
	O…C	13.87 (0.86)	12.78 (0.94)	15.03 (0.90)
	C…P	0.00 (0.00)	0.00 (0.00)	0.00 (0.00)
	P…C	0.59 (0.57)	0.45 (0.94)	1.64 (1.07)
	C…F	0.00 (0.00)	0.00 (0.00)	0.00 (0.00)
	F…C	0.32 (1.14)	0.77 (0.77)	0.75 (1.77)
	N…P	0.00 (0.00)	0.00 (0.00)	0.00 (0.00)
	P…N	0.50 (2.08)	0.28 (2.52)	1.18 (3.30)
	O…P	0.00 (0.00)	0.00 (0.00)	0.00 (0.00)
	P…O	0.19 (0.51)	0.22 (1.33)	0.40 (0.77)
	S…S	0.04 (6.30)	0.04 (6.34)	0.01 (2.09)
	S…P	0.00 (0.00)	0.00 (0.00)	0.00 (0.00)
	P…S	0.02 (2.18)	0.01 (2.90)	0.00 (0.00)
	S…F (S not from Cys)	0.00 (0.00)	0.00 (0.00)	0.00 (0.00)
	F…S (S not from Cys)	0.01 (5.40)	0.01 (1.84)	0.00 (0.00)
	P…P, P…F/F…P and F…F	0.00 (0.00)	0.00 (0.00)	0.00 (0.00)
	**Total**	**42.48**	**40.60**	**40.45**
	**Grand Total**	**100**	**100**	**100**

PL, protein-ligand.

### Polarity ratio and interface atom ratio

Then, we analyzed the polarity ratio (PR), namely the fraction of polar N, O, S atoms at the interface areas of both PP and PL complexes. The interface atom ratio (IR) indicates the fraction of surface atoms that are involved in protein contacts at the interface. As mention before, the interface areas were defined as those residues that are closer than 3 Å (or 4 Å and 5 Å) to at least one residue from the binding partner. Both IR and PR were computed for the datasets of PP and PL complexes from the ABC, PIBASE, and Timbal datasets.

At 3 Å distance threshold, the differences in IR and PR ratios are not representative because only the shortest-distance contacts are considered. For example, when a 3 Å cut-off is used, most carbon atoms are not considered as part of the interfaces as this short distance is shorter than twice the van der Waals radius of carbon (1.7 Å) [38]. Table 6 shows that, as expected, only very small differences were observed when computing PR and IR of PP complexes between the first protein (Pi1) and the second protein (Pi2), as both of them exhibit similar characteristics at binding interfaces. For the larger cut-off distances (4 Å and 5 Å), the polarity ratio (PR) decreases quickly because now all carbon atoms at the surface are included. On the other hand, the interface atom ratio (IR) of 8.0% (4 Å) and 14.0% (5 Å) shows that, expectedly, only a small fraction of the protein surface atoms are included in the interface.

**Table 6:** Interface atom ratio (IR) and polarity ratio (PR) (with standard deviations in parentheses) for interfaces of PP complexes from the ABC and PIBASE datasets.

		PP complexes from the			PP complexes from the		
		ABC dataset			PIBASE dataset		
		Atom distance			Atom distance		
		3Å	4Å	5Å	3Å	4Å	5Å
**IR**	Pi1	0.01 (±0.01)	0.08 (±0.05)	0.14 (±0.08)	0.01 (±0.07)	0.08 (±0.08)	0.13 (±0.09)
	Pi2	0.01 (±0.01)	0.09 (±0.08)	0.15 (±0.13)	0.01 (±0.07)	0.08 (±0.08)	0.13 (±0.10)
**PR**	Pi1	0.87 (±0.22)	0.38 (±0.07)	0.34 (±0.06)	0.72 (±0.20)	0.37 (±0.06)	0.34 (±0.05)
	Pi2	0.85 (±0.22)	0.37 (±0.06)	0.34 (±0.05)	0.71 (±0.20)	0.37 (±0.06)	0.34 (±0.05)

IR, interface atom ratio; PR, polarity ratio; PP, protein-protein; Pi1, first protein; Pi2, second protein.

Table 7 lists the IR and PR ratios of 161 PL complexes from the ABC dataset, 196 PL complexes from the PIBASE dataset, and 89 PL complexes from the Timbal dataset. At the distance threshold of 3 Å, almost no ligands atoms are considered as interfacial atoms whereas the opposite is the case for 5 Å where 93% (PIBASE) and 94% (Timbal) of the ligand atoms are considered as interfacial atoms compared to 78% for ABC. This is suggesting that the PIBASE and Timbal ligands bind more flat on the protein surfaces and/or bind deeper into pockets on the protein surface than the ABC ligands. Finally, the polarity ratios of the proteins in the PL dataset are comparable to the proteins in the PP dataset.

**Table 7:** Interface atom ratio (IR) and polarity ratio (PR) (with standard deviations in parentheses) for interfaces of PL complexes from the ABC, PIBASE and Timbal datasets.

		PL complexes from the			PL complexes from the			PL complexes from the		
		ABC dataset			PIBASE dataset			Timbal dataset		
		Atom distance			Atom distance			Atom distance		
		3Å	4Å	5Å	3Å	4Å	5Å	3Å	4Å	5Å
**IR**	Pi3	0.002 (±0.004)	0.01 (±0.02)	0.03 (±0.03)	0.003 (±0.003)	0.02 (±0.01)	0.03 (±0.02)	0.003 (±0.003)	0.02 (±0.02)	0.03 (±0.03)
	Lj	0.11 (±0.14)	0.55 (±0.27)	0.78 (±0.25)	0.13 (±0.17)	0.74 (±0.25)	0.93 (±0.20)	0.16 (±0.14)	0.75 (±0.21)	0.94 (±0.19)
**PR**	Pi3	0.83 (±0.45)	0.38 (±0.20)	0.35 (±0.14)	0.85 (±0.36)	0.38 (±0.14)	0.34 (±0.12)	0.86 (±0.38)	0.36 (±0.16)	0.32 (±0.12)
	Lj	0.76 (±0.45)	0.38 (±0.23)	0.35 (±0.18)	0.79 (±0.38)	0.33 (±0.17)	0.31 (±0.18)	0.84 (±0.41)	0.38 (±0.25)	0.36 (±0.20)

IR, interface atom ratio; PR, polarity ratio; PL, protein-ligand; Pi3, protein; Lj, ligand.

## Conclusions

In this study, we characterized the residue and atom composition of overlapping protein-protein and protein-ligand interfaces from the ABC and PIBASE databases and compared these to a dataset derived from the Timbal database. According to the statistics, both interface types have, in general, a very similar composition. Among the three datasets of PL complexes, the protein interfaces of the Timbal dataset contain more hydrophobic residues and fewer polar residues than the two other datasets. The ligands in the PIBASE and Timbal datasets bind more flat on the protein surfaces or bind deeper into pockets on the protein surface than ABC ligands. Depending on the respective application in a ligand design project, researchers may consider to bias their principal dataset in one or the other direction. Selecting the appropriate set of reference data may slightly affect the physiochemical characteristics of designed ligands.

## Supporting Information

S1 File Datasets of PP and PL complexes. Dataset of PP complexes from the ABC database **(Table A)**. Dataset of PL complexes from the ABC database **(Table B)**. Dataset of PP complexes from the PIBASE database **(Table C)**. Dataset of PL complexes from the PIBASE database (**Table D**). Dataset of PL complexes from the Timbal database **(Table E)**.
(XLSX)

S2 File Datasets of PP homodimer and heterodimer complexes. Dataset of PP homodimer complexes from the ABC database **(Table A)**. Dataset of PP heterodimer complexes from the ABC database **(Table B)**. Dataset of PP homodimer complexes from the PIBASE database **(Table C)**. Dataset of PP heterodimer complexes from the PIBASE database **(Table D)**.
(XLSX)

S3 File The frequencies of 20 amino acids according to each PDB ID entry in the first protein (Pi1) or second protein (Pi2) of the datasets of PP homodimer/heterodimer complexes and PL complexes. The frequencies of 20 amino acids according to each PDB ID entry in the first protein (Pi1) of the dataset of PP homodimer complexes from the ABC database **(Table A)**. The frequencies of 20 amino acids according to each PDB ID entry in the second protein (Pi2) of the dataset of PP homodimer complexes from the ABC database **(Table B)**. The frequencies of 20 amino acids according to each PDB ID entry in the first protein (Pi1) of the dataset of PP heterodimer complexes from the ABC database **(Table C)**. The frequencies of 20 amino acids according to each PDB ID entry in the second protein (Pi2) of the dataset of PP heterodimer complexes from the ABC database **(Table D)**. The frequencies of 20 amino acids according to each PDB ID entry in the first protein (Pi1) of the dataset of PP homodimer complexes from the PIBASE database **(Table E)**. The frequencies of 20 amino acids according to each PDB ID entry in the second protein (Pi2) of the dataset of PP homodimer complexes from the PIBASE database **(Table F)**. The frequencies of 20 amino acids according to each PDB ID entry in the first protein (Pi1) of the dataset of PP heterodimer complexes from the PIBASE database **(Table G)**. The frequencies of 20 amino acids according to each PDB ID entry in the second protein (Pi2) of the dataset of PP heterodimer complexes from the PIBASE database **(Table H)**. The frequencies of 20 amino acids according to each PDB ID entry of the dataset of protein of PL complexes from the ABC database **(Table I)**. The frequencies of 20 amino acids according to each PDB ID entry of the dataset of protein of PL complexes from the PIBASE database **(Table J)**. The frequencies of 20 amino acids according to each PDB ID entry of the dataset of protein of PL complexes from the Timbal database **(Table K)**.
(XLSX)

S4 File The propensity values of 20 amino acids in the first protein (Pi1) or second protein (Pi2) of the datasets of PP homodimer/heterodimer complexes and PL complexes. The propensity values of 20 amino acids of the first protein (Pi1) of the dataset of PP homodimer complexes from the ABC database (ABC-P1-homo) **(Table A)**. The propensity values of 20 amino acids of the second protein (Pi2) of the dataset of PP homodimer complexes from the ABC database (ABC-P2-homo) **(Table B)**. The propensity values of 20 amino acids of the first protein (Pi1) of the dataset of PP heterodimer complexes from the ABC database (ABC-P1-hetero) **(Table C)**. The propensity values of 20 amino acids of the second protein (Pi2) of the dataset of PP heterodimer complexes from the ABC database (ABC-P2-hetero) **(Table D)**. The propensity values of 20 amino acids of the first protein (Pi1) of the dataset of PP homodimer complexes from the PIBASE database (PIB-P1-homo) **(Table E)**. The propensity values of 20 amino acids of the second protein (Pi2) of the dataset of PP homodimer complexes from the PIBASE database (PIB-P2-homo) **(Table F)**. The propensity values of 20 amino acids of the first protein (Pi1) of the dataset of PP heterodimer complexes from the PIBASE database (PIB-P1-hetero) **(Table G)**. The propensity values of 20 amino acids of the second protein (Pi2) of the dataset of PP heterodimer complexes from the PIBASE database (PIB-P2-hetero) **(Table H)**. The propensity values of 20 amino acids of the dataset of PL complexes from the ABC database (PL-ABC) **(Table I)**. The propensity values of 20 amino acids of the dataset of PL complexes from the PIBASE database (PL-PIBASE) **(Table J)**. The propensity values of 20 amino acids of the dataset of PL complexes from the ABC database (PL-Timbal) **(Table K)**.
(XLSX)

S5 File The frequencies and propensities of 400 amino acid contacts of the datasets of PP complexes. The frequencies and propensities of 400 amino acid contacts of the dataset of PP complexes from the ABC database **(Table A)**. The frequencies and propensities of 400 amino acid contacts of the dataset of PP complexes from the PIBASE database **(Table B)**.
(XLSX)
